# Global patterns of KIR genotype diversity reflect human migration and regional differences

**DOI:** 10.3389/fimmu.2026.1902165

**Published:** 2026-08-12

**Authors:** Shaghik Barani, Lisbeth Guethlein, Derek Middleton, Faviel F. Gonzalez-Galarza, Raja Rajalingam

**Affiliations:** 1 Independent Researcher, Yerevan, Armenia; 2 Immunogenetics and Transplantation Laboratory (ITL), University of California, San Francisco (UCSF), San Francisco, San Francisco, United States; 3 Institute of Infection and Global Health, University of Liverpool, Liverpool, United Kingdom; 4 Center for Biomedical Research, Autonomous University of Coahuila, Torreon, Mexico; 5 School of Engineering and Sciences, Tecnológico de Monterrey, Torreon, Mexico

**Keywords:** evolutionary selection, genetic diversity, killer cell immunoglobulin-like receptors (KIR), KIR genotypes, natural killer cells, NK cell receptors, population genetics

## Abstract

**Background:**

Killer-cell immunoglobulin-like receptors (KIR) are highly polymorphic receptors expressed by natural killer (NK) cells that regulate immune responses through interactions with HLA class I ligands. Variation in KIR gene content influences immunity, reproduction, transplantation outcomes, and susceptibility to disease, yet global patterns of KIR genotype diversity remain incompletely characterized.

**Methods:**

We assembled a curated dataset comprising 25,304 individuals from 192 populations worldwide, representing 662 distinct KIR genotypes. Genotypes were classified according to gene content and haplotype structure, including group A and B haplotype-associated motifs. Population diversity was assessed using Simpson’s diversity index, while geographic distribution, pairwise statistical testing, and principal component analysis were used to evaluate population structure and patterns of genetic variation.

**Results:**

KIR genotype diversity was high overall but showed marked geographic heterogeneity. Populations of Northeast Asia, Southeast Asia and the Americas exhibited reduced diversity due to enrichment of specific genotype subsets, whereas South Asian populations displayed greater diversity and more balanced genotype distributions. Geographic mapping and statistical analyses identified regional differences characterized by distinct KIR genotype profiles. Principal component analysis further demonstrated strong population stratification associated with demographic history and regional differences. The observed distributions could be due to founder effects as well as selection acting on KIR-mediated immune and reproductive functions, maintaining diversity while favoring different genotype subsets in different populations.

**Conclusions:**

This study provides the most comprehensive assessment of global KIR genotype diversity to date and demonstrates how it is possible that human migration, demographic history, and selective pressures have shaped NK cell receptor variation worldwide. These findings advance our understanding of human immunogenetic diversity and have important implications for transplantation, disease association studies, immunotherapy, and the implementation of population-informed precision medicine.

## 1 Introduction

Diversity in immune receptor systems is fundamental to host defense, enabling recognition of a wide spectrum of pathogens and altered self. Central to this process is antigen presentation by human leukocyte antigen (HLA) molecules, which display peptides derived from normal cellular turnover, infection, and cellular stress on the cell surface (1). These peptide–HLA complexes are surveyed by immune cells—including T cells, B cells, and natural killer (NK) cells—through specialized receptors that determine whether tolerance is maintained or an immune response is initiated (2). For T- and B-cell receptors, diversity is generated somatically through V(D)J recombination and mutation during lymphocyte development, allowing individuals with largely identical germline repertoires to generate highly diverse antigen-specific responses (3).

In contrast, NK cell receptor diversity is predominantly germline-encoded and varies between individuals at the population level (4). In humans, this diversity is largely mediated by the killer cell immunoglobulin-like receptors (KIR), a family of receptors that regulate NK cell activity through interactions with HLA class I ligands (5–9). KIR molecules can be either inhibitory or activating, depending on their structural features. Inhibitory KIR possess long cytoplasmic tails containing immunoreceptor tyrosine-based inhibitory motifs (ITIMs), whereas activating KIR have short cytoplasmic tails and signal through adaptor proteins such as DAP12. The balance between these opposing signals determines NK cell responsiveness and plays a critical role in immune surveillance, infection control, and tissue homeostasis.

Both *KIR* and *HLA* exhibit extensive genetic diversity, much of which is shaped by population-specific selective pressures, including pathogen exposure and reproductive constraints (6). The impact of such variation is well established for HLA, where allele frequency differences across populations influence disease susceptibility, transplantation outcomes, and responses to therapy (10, 11). For example, the HLA-B*57:01 allele is strongly associated with hypersensitivity to abacavir, with frequencies varying substantially across populations, thereby affecting treatment strategies (12). The efficacy of emerging immunotherapies, such as the T cell engager tebentafusp, depends on the presence of specific *HLA* alleles (e.g., HLA-A*02:01), whose global frequency distribution constrains therapeutic applicability (13). These examples underscore the importance of understanding population-level variation in immune genes for the development and implementation of precision medicine.

Compared to HLA, global patterns of KIR variation have been less extensively characterized, although increasing attention has been directed toward this locus in recent years (6, 7, 14–17). *KIR* genes encode receptors with either two (2D) or three (3D) extracellular immunoglobulin-like domains and interact primarily with HLA class I molecules, including all HLA-C alleles, subsets of HLA-A and -B, and non-classical molecules such as HLA-G and HLA-F (18, 19). The *KIR* gene cluster is structurally complex, exhibiting variation not only at the allelic level but also in gene content, with individuals differing in the presence or absence of specific KIR genes. Four ‘framework’ genes (*3DL3, 3DP1, 2DL4*, and *3DL2*) are present on nearly all haplotypes (8, 20).

*KIR* haplotypes are broadly classified into group A and group B, which differ in both gene content and functional potential (20, 21). Group A haplotypes have relatively fixed gene content (*2DL1, 2DL3, 2DP1, 3DL1*, and *2DS4*, and the framework genes) and encode predominantly inhibitory receptors. In contrast, group B haplotypes are more variable and include additional activating *KIR* genes, defined by the presence of one or more group B–specific genes (*2DL2, 2DL5, 2DS1, 2DS2, 2DS3, 2DS5*, and *3DS1*) *(*20, 22–27). Recombination between centromeric and telomeric regions further increases haplotypic diversity. This structural and functional variability contributes to substantial differences in *KIR* genotype frequencies across populations.

Variation in *KIR* haplotypes has important biological consequences, particularly in balancing immune defense and reproductive success (6). Group A haplotypes, enriched for inhibitory receptors, have been associated with protection against certain viral infections and malignancies (6, 7, 28–31). However, this advantage may come at a reproductive cost: women homozygous for group A haplotypes who carry fetuses expressing HLA-C2 ligands are at increased risk of pregnancy complications such as pre-eclampsia (6, 32–34). Conversely, the presence of group B haplotypes has been associated with improved reproductive outcomes, although the specific *KIR* genes mediating these effects can differ between populations (32, 33, 35). These findings highlight the role of balancing selection in maintaining KIR diversity.

Beyond their evolutionary and biological significance, KIR are increasingly relevant in clinical contexts (36–39). In hematopoietic cell transplantation, specific combinations of donor KIR and recipient HLA influence relapse risk and survival outcomes, particularly in acute myeloid leukemia (40–43), although these effects are context-dependent (44). KIRs are also emerging as targets in cancer immunotherapy, including checkpoint blockade strategies, with variable efficacy reported across studies (45–47). Notably, genetic variation in KIR, such as KIR3DS1, has been associated with resistance to PD-1 blockade, further emphasizing the clinical relevance of KIR diversity (48). Similarly, individualized KIR-HLA genetic makeup can influence the efficacy of isatuximab immunotherapy in patients with multiple myeloma (49).

Despite these advances, a comprehensive understanding of global *KIR* genotype variation remains incomplete. Existing studies have identified regional differences but are often limited in scale or population representation. To address this gap, we assembled a large, curated dataset of worldwide populations with complete *KIR* genotype data. To maximize global representation, we focused this study on gene content variation as there are a limited number of population studies reporting full genotypes at allele-level resolution. By systematically analyzing genotype frequencies, diversity, and population structure, we aimed to define global patterns of *KIR* variation and to assess how these patterns reflect human migration, demographic history, and potential for selective pressures. This work provides a framework for understanding the role of KIR diversity in human health and disease and for informing future studies in immunogenetics and precision medicine.

## 2 Methods

### 2.1 Dataset construction

A systematic literature search was conducted in PubMed using keywords related to KIR, including KIR receptors, *KIR* genes, *KIR* genotypes, KIR system, KIR family, *KIR* gene variation, KIR diversity, *KIR* gene association, and *KIR* gene content. Duplicate records were removed, and the remaining articles were screened to identify studies reporting *KIR* genotype data from unbiased human population cohorts, i.e. cohorts for which there were no criteria for inclusion beyond membership in the population being sampled. Studies were excluded if they involved clinical trials, randomized controlled trials, or disease-specific cohorts as these disease associated cohorts could have bias introduced by selective sampling. In addition, systematic reviews, meta-analyses, non-human subjects, non-English publications, conference abstracts, or studies lacking complete genotype data were also excluded as they contained either duplicated, irrelevant or incomplete data.

In parallel, *KIR* genotype data were obtained from the Allele Frequency Net Database (AFND) (50). Only populations with complete genotype reporting at the gene content level were included. The datasets derived from PubMed and AFND were compared to assess overlap. Of the final dataset, 144 populations were identified by both sources, 47 were unique to AFND, and one population was identified only through the PubMed literature search. This resulted in a final dataset of 192 populations with 662 distinct genotypes from 25,304 individuals. Both databases were accessed on February 1, 2026.

### 2.2 Genotype classification

*KIR* genotypes were first classified as AA or Bx based on gene content. Genotypes containing only the canonical group A genes (*3DL3, 2DL1, 2DL3, 2DL4, 3DL1, 2DS4*, and *3DL2*) were designated as AA, whereas genotypes containing at least one group B–specific gene were classified as Bx. To further characterize structural variation within the KIR locus, genotypes were assigned centromeric (Cen) and telomeric (Tel) motifs. These regions are separated by a recombination hotspot and can vary independently. The following criteria were used:

1. Cen AA: at least one of *2DL1* or *2DL3*, and no *2DL2, 2DS2*, or *2DS3*.
2. Cen BB: at least one of *2DL2, 2DS2*, or *2DS3*, and no *2DL1* or *2DL3*.
3. Cen AB: at least one of *2DL1* or *2DL3*, and at least one of *2DL2, 2DS2*, or *2DS3*.
4. Tel AA: at least one of *3DL1* or *2DS4*, and no *3DS1, 2DS1*, or *2DS5*.
5. Tel BB: at least one of *3DS1, 2DS1*, or *2DS5*, and no *3DL1* or *2DS4*.
6. Tel AB: at least one of *3DL1* or *2DS4*, and at least one of *3DS1, 2DS1*, or *2DS5*.

To further stratify Bx genotypes, we categorized them based on the presence of defined centromeric and telomeric group B gene clusters, as previously described (51). Genotypes were classified as C4 if they contained all four centromeric genes (*2DS2, 2DL2, 2DS3*, and *2DL5*), and as Cx if one or more of these genes were absent. Similarly, genotypes were classified as T4 if they contained all four telomeric genes (*3DS1, 2DS5, 2DL5*, and *2DS1*), and as Tx if one or more of these genes were absent. Based on these designations, genotypes were grouped into five categories: *AA, CxTx, C4Tx, CxT4*, and *C4T4*.

### 2.3 Geographic mapping

Geographic coordinates for each population were obtained from AFND or the corresponding publication. Maps were generated using QGIS (version 3.40.15). Genotype frequencies were visualized using a standardized color scale ranging from 0 to 1 in increments of 0.1, applied consistently across all maps. For **Supplementary Figure 1**, population markers were replaced with pie charts to represent the relative frequencies of genotype subsets.

### 2.4 Diversity analysis

Genotype diversity was quantified using Simpson’s index of diversity (1 − D) (52). Diversity was calculated for each population individually and for aggregated regional subgroups. Analyses were performed both at the level of individual genotypes and for grouped categories (AA and Bx subsets). The Simpson’s Diversity index was calculated using the formula:


$$1-\frac{\sum n\left(n-1\right)}{N\left(N-1\right)}$$

where *n* represents the number of individuals with a given genotype, and N is the total number of individuals in the population.

### 2.5 Statistical analysis

Principal component analysis (PCA), chi-square analysis, pairwise testing of frequency distributions, and heatmap visualization were performed in R (version 4.5.1) (53). Heatmap visualizations were generated using ggplot2 (54). Statistical analyses were performed using base R. PCA was performed using the psych (55), dplyr (56), and corrr (57) packages. Rscripts are available from the authors upon request. Simpson’s diversity index was calculated in Microsoft Excel.

## 3 Results

### 3.1 Construction of a global KIR genotype dataset

**Figure 1:**
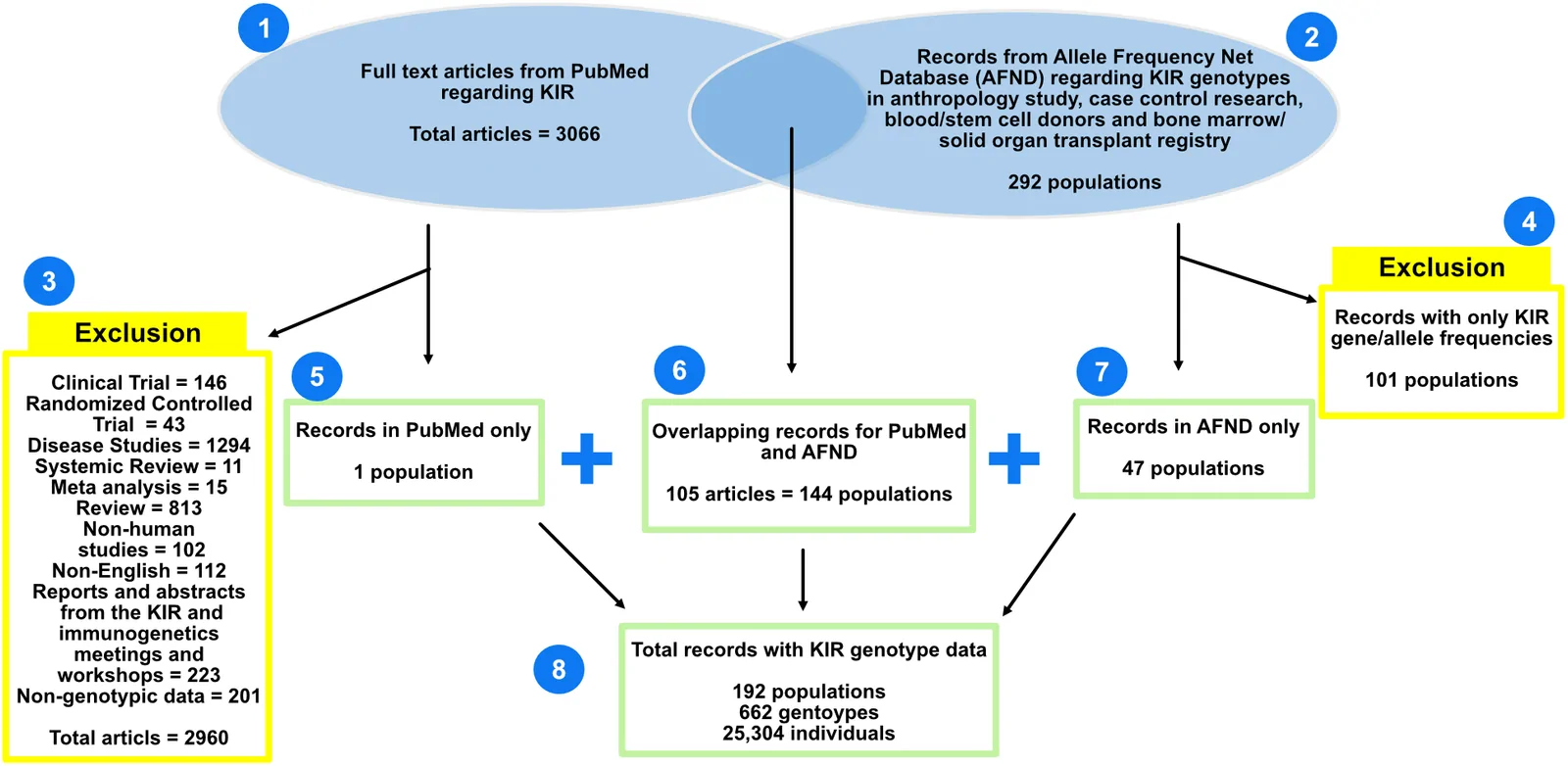
Dataset construction flowchart. PubMed was queried for articles related to KIR, and population-level *KIR* genotype data were collected from the Allele Frequency Net Database (AFND) (blue ovals). Records and populations that did not meet the selection criteria were excluded (yellow boxes), resulting in the final dataset. Comparison of populations identified through the literature search (green boxes) and AFND showed that 144 populations were present in both sources, one population was identified only through PubMed, and 47 populations were unique to AFND. The final dataset comprised 192 populations with 662 unique genotypes from 25,304 individuals.

A comprehensive dataset of *KIR* genotype frequencies was assembled from published studies and the Allele Frequency Net Database (AFND) (50), as outlined in **Figure 1**. This study is focused on genotypes at the gene content level to attain the greatest global representation. At present there are only a limited number of populations with complete genotype information at the allele level. While limiting the study to gene content removes identification of potential functional variability encoded by specific alleles it allowed for a broader representation of global populations. Following systematic screening and application of exclusion criteria, only populations with complete and unbiased genotype data were retained. Comparison of datasets identified 144 populations common to both sources, one population unique to the PubMed literature search, and 47 populations unique to AFND. The final dataset comprised 192 populations, representing 25,304 individuals and 662 distinct *KIR* genotypes.

**Figure 2:**
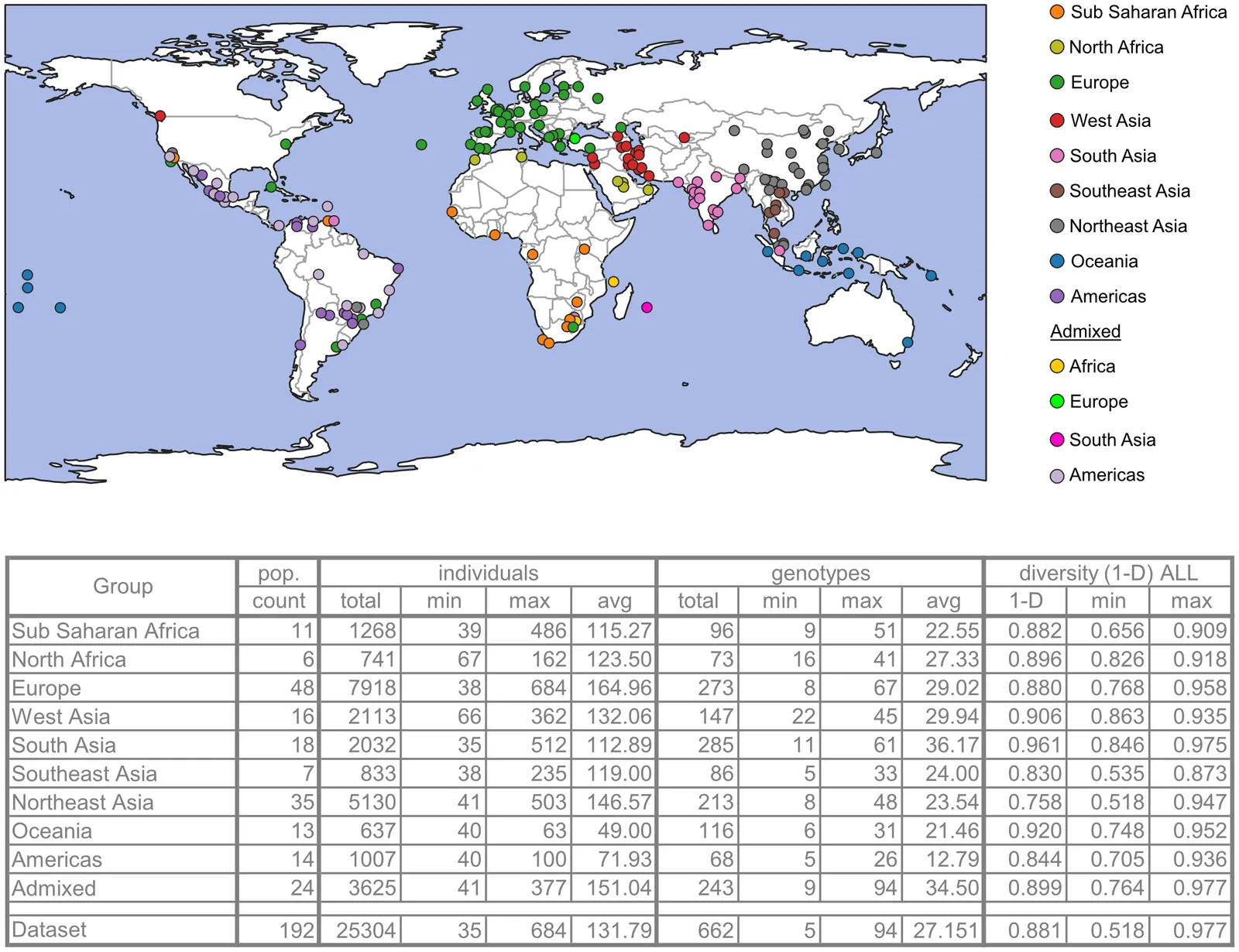
Worldwide distribution of populations analyzed. Geographic locations of populations included in the dataset are shown as filled circles, with colors representing region of origin. To improve visualization of multiple populations at the same location (e.g., Singapore, South Africa, and California, USA), a small positional offset was applied, leading to overlapping circles. The table below the map summarizes the population counts per regional group, along with the minimum, maximum, and average population sizes. It also includes the number of genotypes per region (minimum, maximum, and average) and the Simpson diversity index calculated for each region, with corresponding ranges for individual populations. A complete list of populations, abbreviations, and references is provided in **Supplementary Table 1**. Population-level data (coordinates, genotype and gene frequencies, and diversity values) are available in **Supplementary Table 2**, and region-level summaries are provided in **Supplementary Table 3**.

The global distribution of these populations is shown in **Figure 2** (upper panel). Populations were categorized into 9 geographic groups, indicated by color coding. Admixed populations were analyzed separately and are indicated by distinct coloring. Recently migrated populations were classified according to their reported region of origin; for example, Japanese populations in Brazil were categorized as Northeast Asia (gray), and South Asian (pink) and European (green) populations in South Africa retained their respective designations. The table in **Figure 2** (lower panel) summarizes the number of populations per region, along with their size range and average sample size. A complete list of populations and references is provided in **Supplementary Table 1**.

### 3.2 Global patterns of KIR genotype diversity

The table in **Figure 2** also shows the number of genotypes in each region, along with the minimum, maximum, and average numbers of genotypes within that region. Substantial variation in *KIR* genotype diversity was observed across populations. The number of genotypes per population ranged widely, with the highest averages observed in South Asian (36.17) and admixed populations (34.5). In contrast, populations of the Americas exhibited the lowest diversity, averaging 12.79 genotypes per population.

To account for differences in sample size and the influence of rare genotypes, diversity was further assessed using Simpson’s index. South Asian populations exhibited the highest overall diversity (0.961), followed by Oceanian (0.920) and West Asian (0.902) populations. Despite having the fewest genotypes per population, American populations showed moderate diversity (0.844), whereas Northeast Asian populations had the lowest diversity (0.758), despite a relatively higher average number of genotypes (23.54). These findings indicate that genotype distribution, rather than absolute genotype count, drives overall diversity.

### 3.3 Stratification of genotypes by B-haplotype gene content

**Figure 3:**
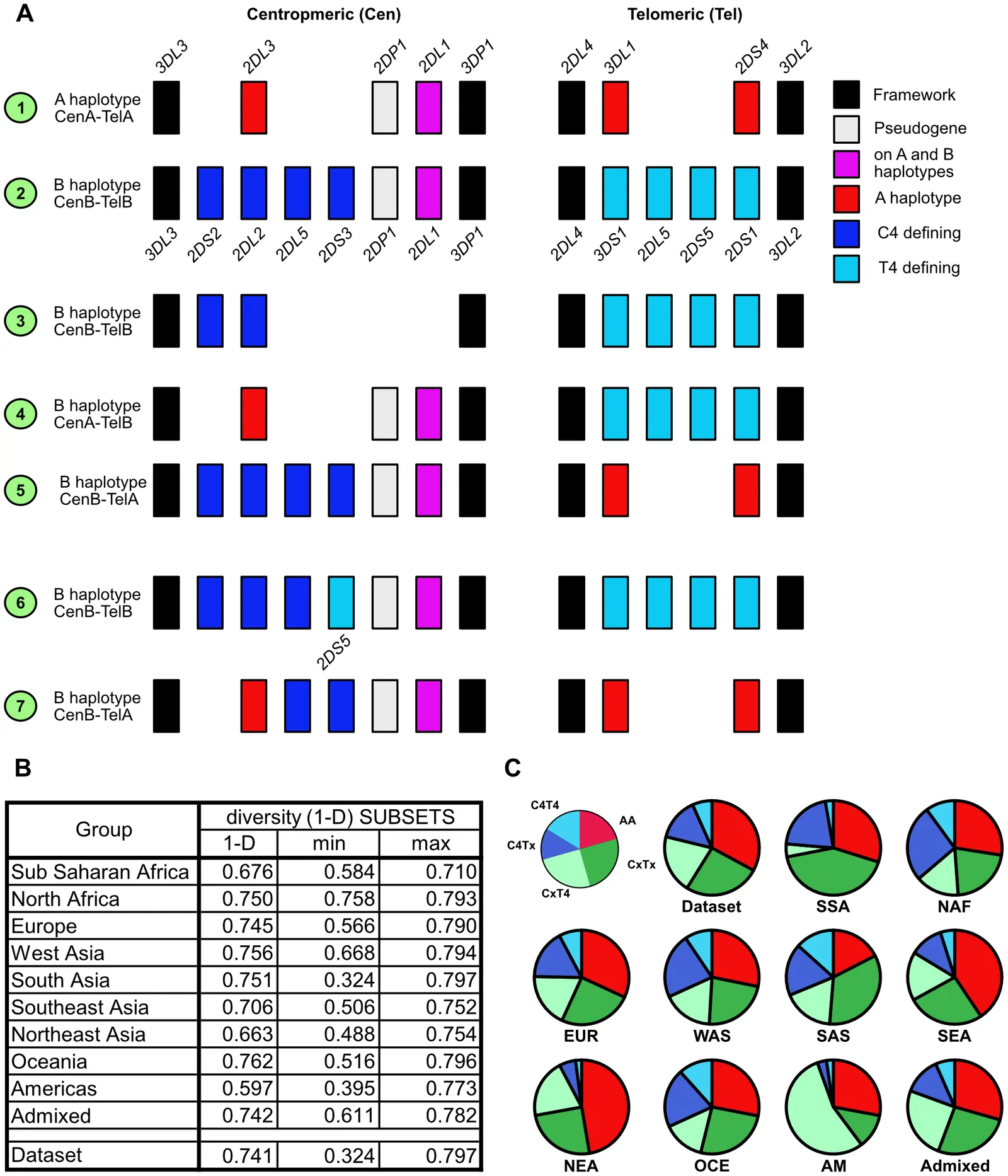
KIR haplotype organization and definition of C4 and T4 gene clusters. **(A)** Schematic representation of *KIR* haplotype structures based on fully sequenced haplotypes reported by Pyo et al. (25) Numbers in green circles are included for reference. Framework genes present on all haplotypes (with rare exceptions) are shown as black squares, and pseudogenes are shown as white squares. Red squares indicate genes defining group A haplotypes, along with *KIR2DL1* (purple square), which is present in both group A and group B haplotypes. Group B–specific genes are indicated in blue. Presence of the four genes indicated by dark blue squares (*2DS2, 2DL2, 2DS3*, and *2DL5*) defines the C4 cluster, while presence of the four genes indicated by light blue squares (*3DS1, 2DS5, 2DL5*, and *2DS1*) defines the T4 cluster. Genotypes were first classified as *AA* or *Bx*, and *Bx* genotypes were further evaluated for the presence of *C4* and/or *T4*. Absence of these clusters is denoted as *Cx* or *Tx*, respectively. **(B)** Diversity values for regional subgroups based on *AA/Bx* subset frequencies. **(C)** Pie charts showing the distribution of genotype subsets across regions.

To further investigate patterns of diversity, *KIR* genotypes were classified into five subgroups based on the presence of group B haplotype–specific gene clusters (**Figure 3A**) (25). *KIR* haplotypes are broadly divided into groups A and B, with group B defined by the presence of one or more specific genes (*2DL2, 2DL5, 2DS1, 2DS2, 2DS3, 2DS5*, and *3DS1*). These haplotypes diversify through recombination and structural variation. KIR A haplotypes encode mostly inhibitory KIR whereas KIR B haplotypes encode varying numbers of activating KIR.

As described previously (51), we defined the “B-ness” or activating potential of *KIR* genotypes based on the presence or absence of two gene clusters shown in **Figure 3A**, referred to as the “C4-defining” and “T4-defining” clusters. Genotypes were first classified as *AA* or *Bx*, and *Bx* genotypes were then further subdivided into four groups—*CxTx, CxT4, C4Tx*, and *C4T4*—based on the presence or absence of the C4 and/or T4 gene clusters. This classification resulted in a more balanced distribution of genotype classes across regional groups. C4-positive genotypes could arise either from the presence of all four genes on a single haplotype (e.g., haplotypes 2 or 5 in **Figure 3A**) or from the combination of two haplotypes (e.g., haplotypes 3 and 7 in **Figure 3A**). This classification provides a functional framework for comparing populations. These groupings also define haplotypes with greater or lesser potential for encoding activating KIR. AA genotypes encode the fewest with the only activating KIR encoded being 2DS4 in the telomeric region. C4T4 genotypes encode the maximum number of activating KIR genes, C4Tx and CxT4 an intermediate number and CxTx the fewest, but still more than the AA genotype. We focused on this categorization as it considered the complete genotype rather than assessing the centromeric and telomeric regions independently.

Reanalysis of diversity using these grouped categories revealed distinct patterns (**Figures 3B, C**). Oceanian populations exhibited the highest diversity (0.762), reflecting a relatively balanced distribution across genotype subsets (**Figure 3C**). In contrast, American (0.597) and Northeast Asian (0.663) populations showed lower diversity, driven by the predominance of a single subset—*CxT4* in American and *AA* in Northeast Asian populations. Diversity in the Sub-Saharan African populations was also lower, 0.676, due to the very low frequency of T4 containing genotypes (**Figure 3C**).

### 3.4 Distribution of common KIR genotypes

**Figure 4:**
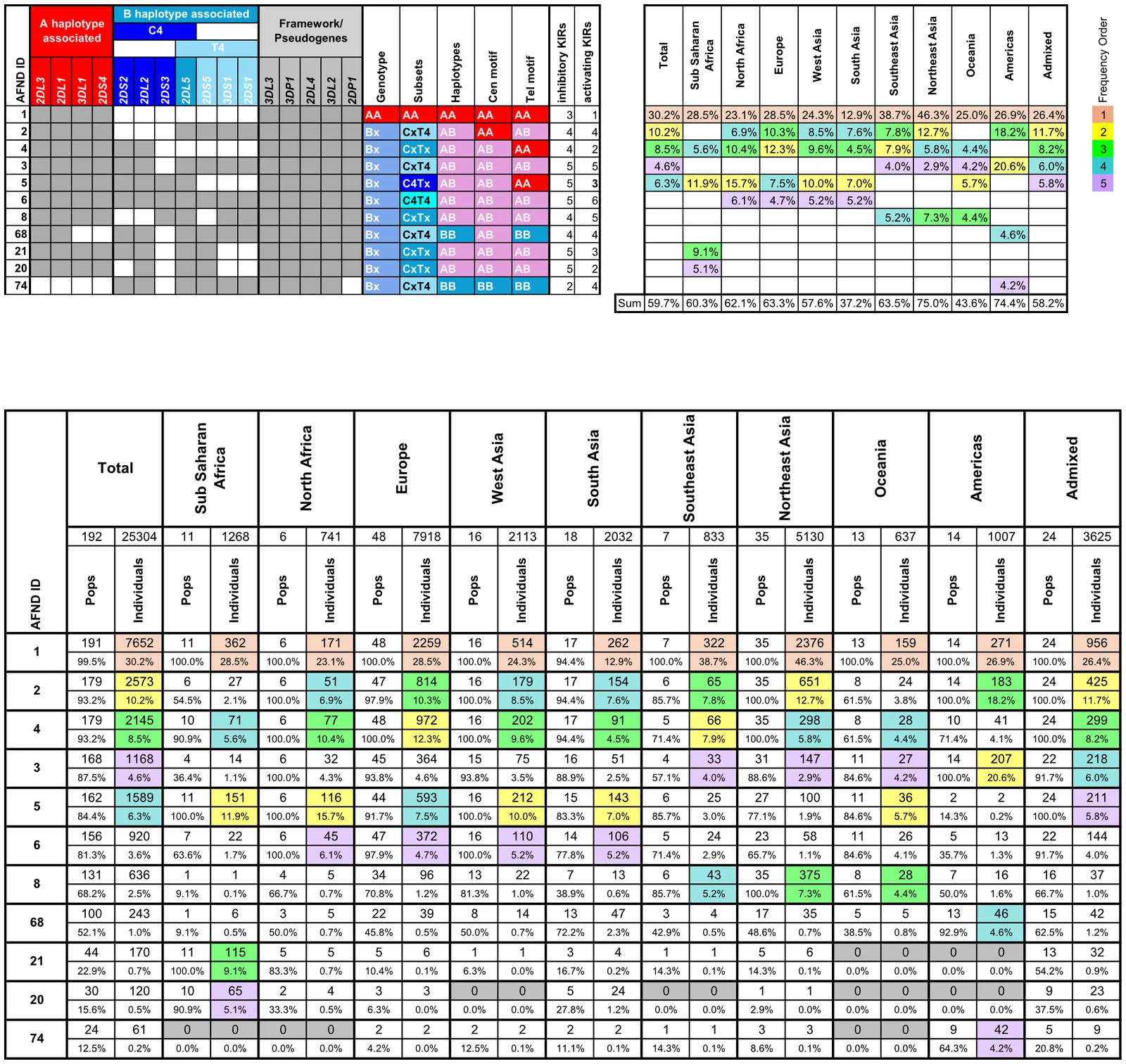
Most frequent KIR genotypes worldwide and by region. The upper panel shows genotypes ranked among the top five by frequency in at least one geographic region, identified by their AFND ID numbers. Shaded boxes indicate the presence of a gene. Genotypes are annotated by subset classification, inferred haplotypes, centromeric and telomeric motifs, and the number of activating and inhibitory KIR. Color coding highlights genotype categories (*AA* in red, *AB* in purple, and other *B* genotypes in blue). To the right, the top five genotypes for each region are shown with their frequencies. The most frequent genotype is coded in orange, followed by yellow, green, blue, and purple, in rank order two through five. The lower panel shows complete frequency data, including the proportion of populations within each region carrying each genotype and the overall frequency. Full genotype distributions by population are provided in **Supplementary Table 2**, and region-level summaries are available in **Supplementary Table 3**.

We next examined the distribution of the most frequent genotypes across populations (**Figure 4**). The top five genotypes for each region were identified, along with their AFND IDs, inferred haplotypes, centromeric and telomeric motifs, and functional characteristics.

The *AA* genotype was the most frequent genotype across all regions (**Figure 4**). Its frequency ranged from 12.9% in South Asian populations to 46.3% in Northeast Asian populations. Many regions exhibited unique combinations of their top five genotypes. However, despite this apparent uniqueness when considering only the most frequent genotypes, for example, AFND ID 68 in America, most haplotypes are found worldwide, albeit at lower frequencies (**Figure 4**, lower panel). Notably, several regions, including North Africa, Europe, West Asia and South Asia, shared the same top five genotypes (AFND IDs 1, 2, 4, 5, and 6), although their relative frequencies differed. The contribution of the five most frequent genotypes to the overall genotype distribution varied substantially across populations, ranging from 37.2% to 7.5%, reflecting differences in population structure and genetic diversity.

### 3.5 Geographic distribution of genotype subsets

**Figure 5:**
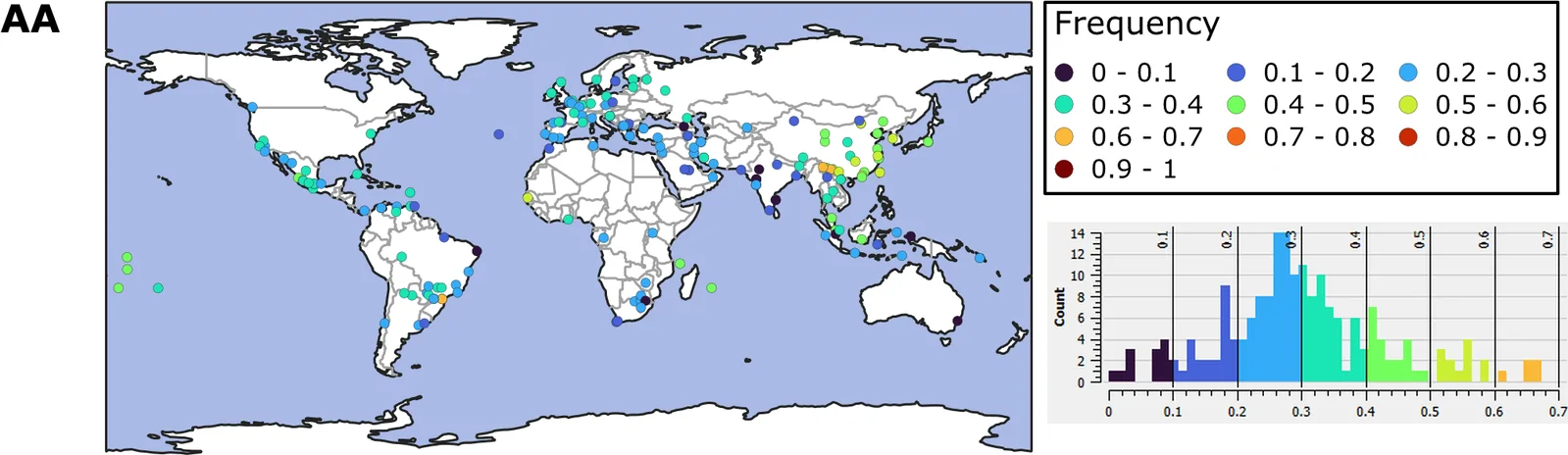
Worldwide distribution of *KIR AA* genotypes. The populations shown in **Figure 2** are color-coded by *AA* genotype frequency, with the legend shown on the right. The histogram in the lower right displays the distribution of *AA* genotype frequencies across populations, with frequency on the x-axis and number of populations on the y-axis. Histogram colors correspond to the map color scale.

**Figure 6:**
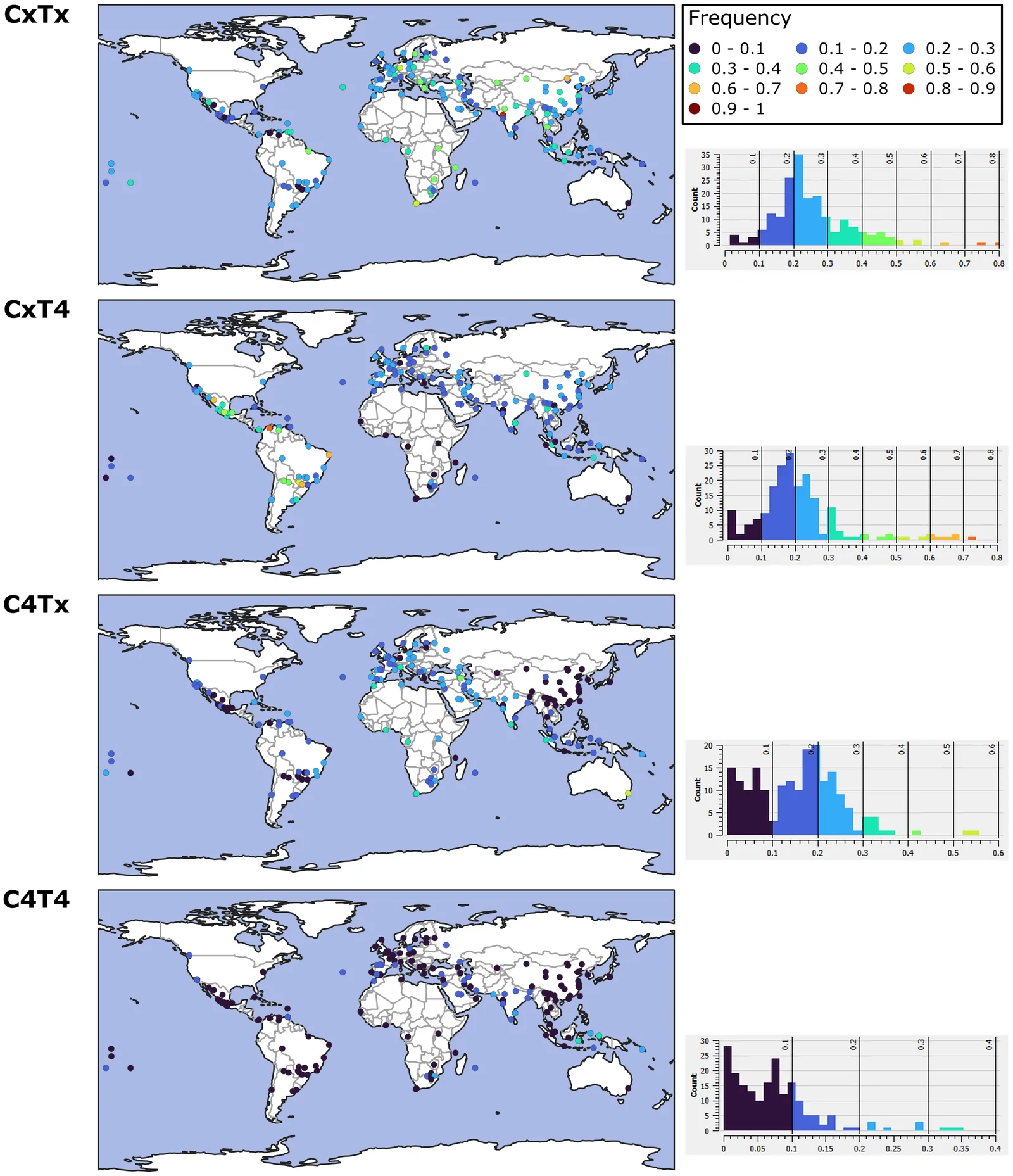
Worldwide distribution of *KIR Bx* genotype subsets. *Bx* genotypes were subdivided into four categories (*CxTx, C4Tx, CxT4*, and *C4T4*) based on the presence or absence of *C4* and *T4* gene clusters defined in **Figure 3**. The frequency of each subset is shown on the world map using the same color scale as in **Figure 5**. A composite map summarizing all genotype frequencies is provided in **Supplementary Figure 1**.

We mapped the frequencies of genotype subsets to evaluate their geographic distribution (**Figures 5**, **6**). AA genotypes were most frequent in Northeast and Southeast Asian populations, with the lowest frequencies observed in South Asia and intermediate levels elsewhere (**Figure 5**). Among the Bx subsets (**Figure 6**), distinct regional patterns were evident. *CxTx* genotypes were enriched in Africa and parts of Europe, South Asia, and East Asia. In contrast, *CxT4* genotypes were most frequent in the Americas and were largely absent in African and some Pacific Island populations. Conversely, *C4Tx* genotypes were most common in African populations and were largely absent in East Asia and the Americas. *C4T4* genotypes were generally rare worldwide but occurred at relatively higher frequencies in South Asian and some Oceanian populations. A global summary of genotype frequencies is provided in **Supplementary Figure 1**. Detailed genotype data for all populations are available in **Supplementary Table 2**, and genotype frequencies and diversity estimates for regions are presented in **Supplementary Table 3**.

### 3.6 Regional differences in KIR genotype frequency visualized by heatmap

**Figure 7:**
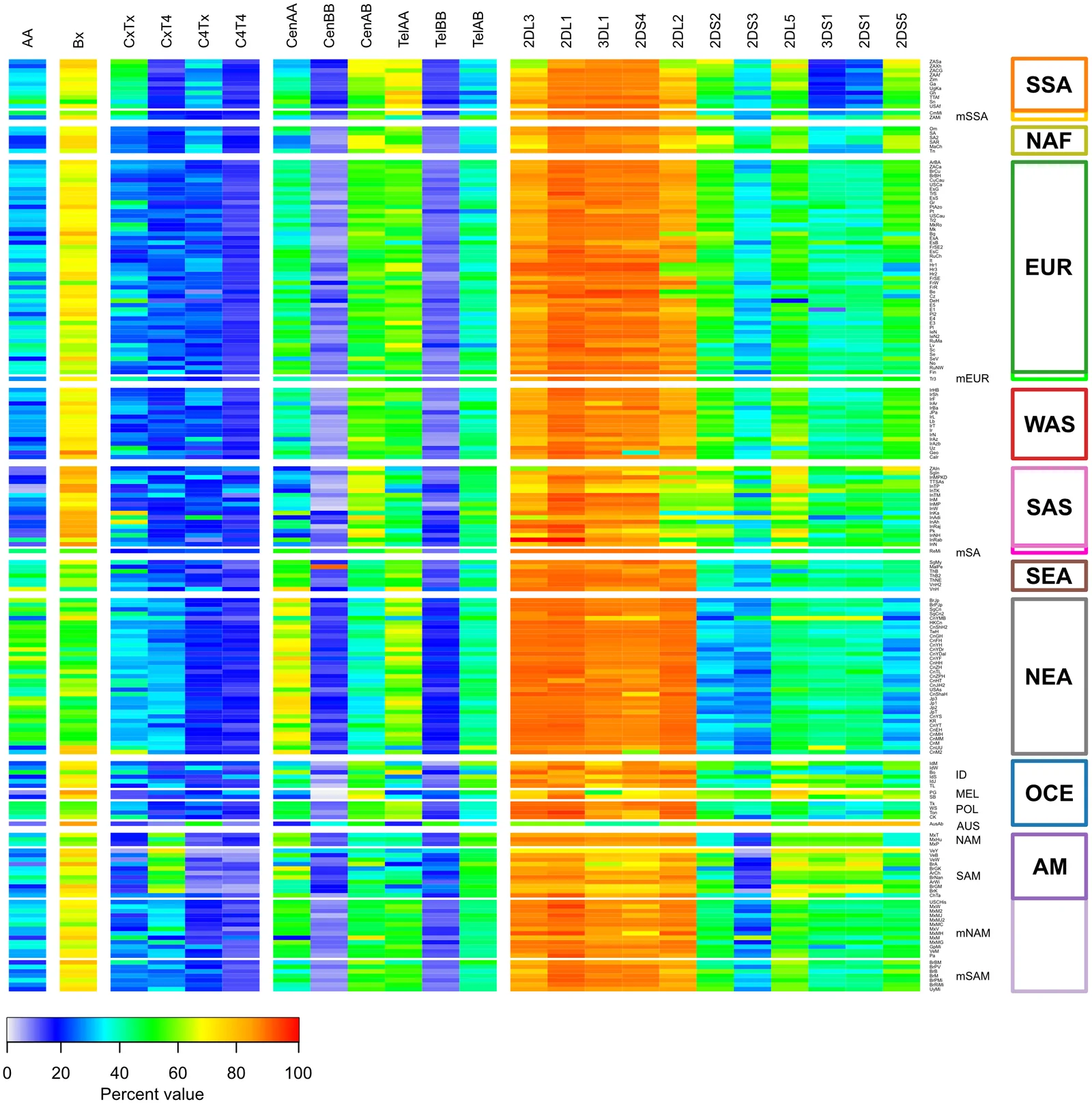
Visualization of regional and population frequency differences. Populations are displayed by region with admixed populations separated. Within regions populations were sorted by latitude then longitude. The region identifiers are shown at the right (Sub-Saharan Africa, SSA; North Africa, NAF; Europe, EUR; West Asia, WAS; South Asia, SAS; Southeast Asia, SEA; Northeast Asia, NEA; Oceania, OCE; Americas, AM). For the Americas and Oceania subgroups of populations are indicated (OCE: Indonesia, Id; Melanesia, MEL; Polynesia, POL; Australia, AUS and AM: North America, NAM; South America, SAM). From left to right frequencies are shown for AA, Bx, the four Bx subgroups defined by presence absence of C4 and T4 clusters, Cen and Tel groups and individual genes grouped by A defining (2DL3 to 2DS4) or B specific (2DL2 to 2DS5).

**Figure 7** shows a heatmap of genotype and gene frequencies for the populations in this study. It is organized by regions, with admixed populations of the region separated. Within each region, populations were organized by latitude then longitude to provide some geographic context. Apparent from the heatmap are qualitative differences both within and between regions although some regions appear to have stronger regional identity.

As shown in **Figure 6**, the Sub-Saharan populations are characterized by an elevation of CxTx genotypes and very low frequencies of T4 containing haplotypes (**Figure 7**). The lack of T4 genotypes is consistent with the very low frequency of 3DS1 and 2DS1 in these populations. This pattern is not seen in North African populations that appear similar in heatmap patterning to the European, West Asian and South Asian populations. The only apparent difference is the overall higher AA genotype frequency in the European populations.

Within the European populations there is variation in the Bx subgroups with select populations showing elevation of the CxTx genotype. This elevated frequency is not specific to a certain subregion with the populations showing the elevation being found as distant as Greece, Sweden and the Azores. The South Asian populations also show variation in frequency although here the differences seem to be largely between northern and southern populations for CxTx frequency. The South Asian populations overall have very low frequencies of AA genotypes compared to other populations (**Figure 5**) and the heatmap shows a more even distribution of genotypes in the Bx subgroups for the more southern populations whereas the northern populations have a preponderance of CxTx genotypes (**Figure 7**).

The populations of Northeast and Southeast Asia had the highest overall AA genotype frequency as shown in **Figure 5**. Despite the small number of populations sampled, distinct patterns could be observed for subgroups of the Oceanian populations. In particular, Polynesian populations had elevated frequencies of AA genotypes compared to the others. Finally, the populations of the Americas showed a marked increase in the frequency of the CxT4 genotype as was seen in **Figure 6**.

### 3.7 Confirmation of regional difference through principal component analysis and pairwise testing

**Figure 8:**
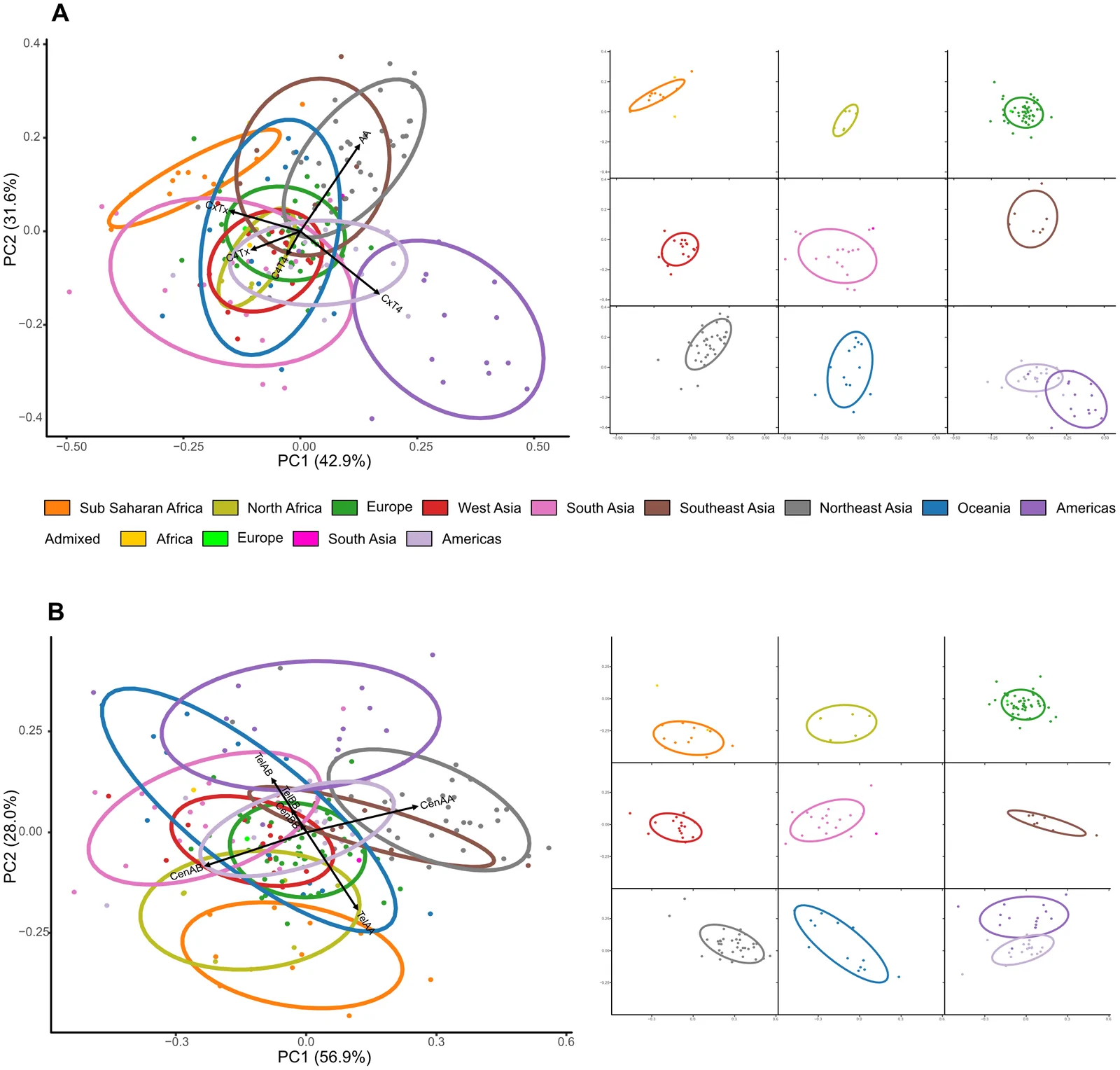
Principal component analysis of population clustering. **(A)** The left panel shows the PCA analysis for AA, CxTx, CxT4, C4Tx and C4T4 with populations and clusters color coded by region. Ellipses represent one standard deviation from the mean of the group. The right panel shows each regional group separately for improved visualization, with points positioned identically to the main PCA plot. Additional PCA details including the contribution and composition of each component and the contribution of each variable to the plot are provided in **Supplementary Figure 2**. **(B)** Shows the PCA analysis using the Cen and Tel genotypes. Color coding as above and additional details in **Supplementary Figure 2**.

Principal component analysis (PCA) (**Figure 8**) supported the observations from the heatmap (**Figure 7**). Northeast and Southeast Asian populations clustered along the *AA* axis, populations from the Americas formed a distinct cluster along the *CxT4* axis, and Sub-Saharan populations clustered along the *CxTx* axis (**Figure 8A**, left panel). Other populations occupied more central positions in the plot, reflecting more balanced genotype distributions. Isolation of the region-specific clusters (**Figure 8A**, right panel) revealed differences in within-group variability; for example, European populations formed a tight cluster, whereas South Asian populations showed greater dispersion, indicating higher internal diversity.

As a comparison, we also performed PCA analysis using the Cen and Tel genotype frequencies (**Figure 8B**). Similar to the PCA results in **Figure 8A** the Sub-Saharan African, American and Northeast and Southeast Asian clusters were distinct. The South Asian cluster was slightly offset and broad reflecting the variability of the populations with the region. Central to the plot were the Oceania cluster which was broadly spread and overlapping with the European and West Asian clusters. Distinct in the plot of Cen and Tel genotype frequencies is the distinction of the North African population cluster which is overlapping with the European and West Asian clusters in the analysis in **Figure 8A**. This is possibly due to the decrease in Cen AA and increases observed for Cen AB and Tel AA in select North African populations. Additional PCA results including the contribution of individual components and the composition of the components are provided in **Supplementary Figure 2**.

**Figure 9:**
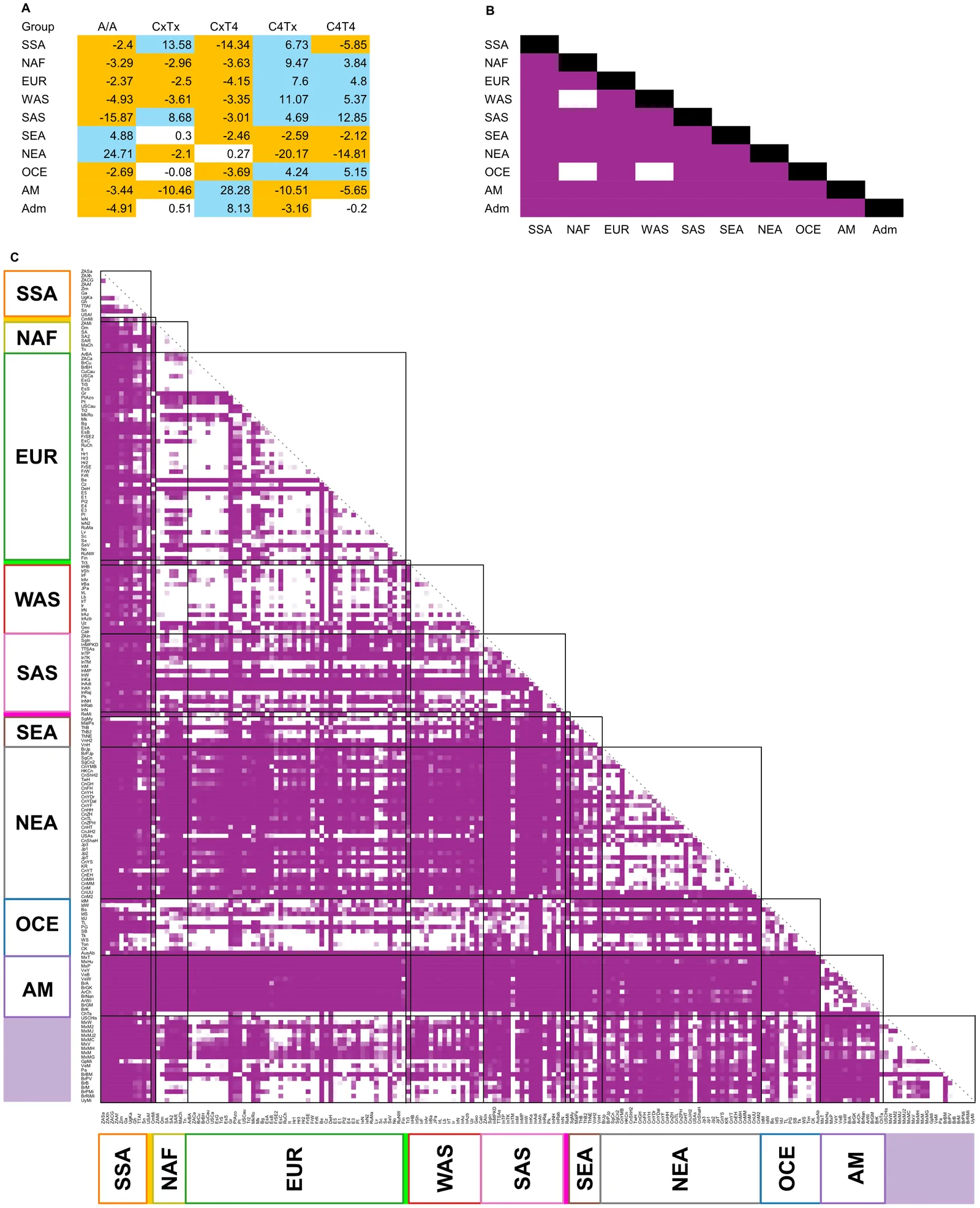
Statistical analysis of regional and population differences. **(A)** Shows the result of a chi-square analysis of differences in frequency distribution of AA, CxTx, CxT4, C4Tx and C4T4 genotypes in regional groups. All non-admixed populations within a group were combined for this analysis. The result was significant with χ^2^ = 2801.9, df = 36, p-value< 2.2e-16. Gold shading indicates values of less than -1.96 indicating significantly fewer individuals than expected (p< 0.05). Blue shading indicates values of greater than1.96 indicating significantly more individuals than expected (p< 0.05). **(B)** shows the pairwise-comparison between regional groups. Purple squares indicate p< 0.05. **(C)** shows the pairwise-comparison of individual populations ordered by regional groups. Squares are shaded by p-value with white indicating p > 0.05 and darker shades of purple indicating higher significance. A complete list of pairwise-comparison p-values is in **Supplementary Table 4**.

A Chi-square analysis was performed to test the differences between the major geographic groups and the result was highly significant (χ^2^ = 2801.9, df = 36, p-value< 2.2e-16, **Figure 9A**). The results confirm the elevation or depression of genotype frequencies that was shown qualitatively in the heatmap (**Figure 7**) and described above. *Post hoc* pairwise comparisons using Fisher’s exact test were then performed to test differences between the groups. All regions showed significant difference with the exception of North Africa, West Asia and Oceania which did not differ at p< 0.05 (**Figure 9B**; exact p-values in **Supplementary Table 4**).

Finally, a Fisher’s test with *post hoc* pairwise analysis was performed for all populations individually. The results are shown in **Figure 9C** with significance values shaded. The populations of the Americas show limited significant differences within the region but are significantly different from essentially all populations outside of the region consistent with the heatmap observations and the distinct clustering in the PCA analysis. Similarly, the Sub-Saharan African populations despite having slightly more internal differences are distinct from most other populations again consistent with the heatmap and PCA. The North African populations have few internal differences as well as showing few differences with select populations from Europe, West Asia and Oceania. The European populations show some internal differences with the South Asian populations having the most significant differences. Finally, Northeast Asian populations have some internal differences and are largely distinct from other world populations.

## 4 Discussion

In this study, we present a comprehensive analysis of global *KIR* genotype diversity by integrating data from 192 populations spanning diverse backgrounds. This dataset includes both indigenous populations and those with more recent migration histories, providing a broad snapshot of contemporary *KIR* genotype frequency patterns worldwide. Although population representation is not uniform - due to limitations in available data and the exclusion of potentially biased cohorts, such as disease-specific studies-the dataset provides sufficient coverage to identify major global trends and population-level differences in KIR variation. The dataset was also focused on genotype differences at the level of gene content in order to maximize world coverage. As sufficient genotype data at the allele level becomes available it will be of interest to revisit this type of analysis.

Overall, *KIR* genotypic diversity was high across populations, consistent with previous studies (6, 7, 14, 15, 17, 58). However, this diversity was not evenly distributed. When genotypes were grouped into functionally relevant subsets (*AA* and *Bx*-derived categories), distinct patterns emerged. Populations from South Asia, West Asia and Oceania exhibited high diversity, reflecting relatively balanced distributions of genotype subsets. In contrast, American, Northeast Asian, and Sub-Saharan African populations showed reduced diversity, driven by the predominance of specific genotype subsets. In American populations, this was largely due to enrichment of the *CxT4* subset (approximately 55%), while Northeast Asian populations were characterized by high frequencies of *AA* genotypes (approximately 47%). Sub-Saharan African populations showed enrichment of the *CxTx* subset (approximately 43%). These patterns were reflected in both Simpson’s diversity index and clustering analyses, indicating that diversity is driven more by genotype distribution than by the absolute number of genotypes.

The geographic distribution of genotype subsets further highlights the strong regional structuring of KIR variation (17). The enrichment of *AA* genotypes in Northeast and Southeast Asian populations and *CxT4* genotypes in the American populations represent clear signatures of population history. The reduced diversity observed in American populations is consistent with founder effects and bottlenecks associated with migration into the Americas, whereas the higher diversity observed in South Asian populations likely reflects long-term population size, admixture, and complex demographic history. Similarly, the predominance of *Bx* subsets in African populations underscores the distinct genetic architecture of KIR in these populations.

Principal component and statistical analyses reinforce these findings by identifying significant differences between regional population groups. The pairwise statistical analysis of individual populations demonstrates that there is intraregional variation and there are instances where populations may be more similar to those found in another region. These patterns suggest that shared ancestry and possibly convergent selection contribute to the observed clustering.

Although tests for selection were not performed on this dataset, the observed distribution of *KIR* genotypes could reflect the combined effects of demographic history (including founder effects and drift) and selective pressures acting on immune and reproductive functions. KIR–HLA interactions play critical roles in both antiviral immunity and placental development, and selection acting on these processes can lead to opposing outcomes (6). Group *A* haplotypes, enriched for inhibitory receptors, have been associated with improved control of viral infections and certain malignancies (6, 7, 28–31), whereas group *B* haplotypes, which include activating receptors, have been linked to improved reproductive success, particularly in the context of maternal–fetal interactions involving HLA-C (32, 33, 35). While some of the populations that are part of this dataset have companion HLA data, this was not available for all populations, and a study of co-evolution was not undertaken. As additional high-resolution KIR and HLA data become available for world populations a study of their co-evolution will be of interest.

Evolutionary processes, including recombination and introgression, have also contributed to shaping KIR diversity. The low frequency of certain telomeric *B* haplotype components, such as those associated with the T4 group, in African populations—and their higher frequency outside Africa—is consistent with proposed introgression of KIR variants, including KIR3DS1, from archaic human populations following migration out of Africa (59). Such events may have introduced novel variants that conferred advantages in new environments, further contributing to the global diversity of *KIR* genotypes.

From a clinical perspective, the substantial variation in KIR genotype frequencies across populations has important implications. In hematopoietic cell transplantation, specific KIR–HLA combinations can influence relapse risk and survival outcomes, particularly in acute myeloid leukemia (40–42), although these effects are context-dependent. Similarly, KIR variation has been implicated in differential responses to cancer immunotherapies, including checkpoint blockade. The marked population differences observed in this study suggest that the effectiveness of such therapies may vary globally, underscoring the importance of incorporating population-specific immunogenetic data into clinical study design and therapeutic development.

Despite the strengths of this study, including the dataset’s size and diversity, several limitations should be considered. Population representation remains uneven, with some geographic regions underrepresented. In addition, the analysis is based on genotype-level data and does not capture allelic variation, which may further influence functional outcomes. Although efforts were made to exclude biased cohorts, subtle sampling biases may persist. Future studies incorporating high-resolution allele-level data, functional assays, and more comprehensive population sampling will be important to refine these findings.

In summary, this study provides a detailed characterization of global KIR genotype diversity and demonstrates how demographic history and selective pressures may have shaped the distribution of immune receptor variation. These findings enhance our understanding of population differences in immunity, reproduction, and disease susceptibility and provide a foundation for future research in immunogenetics and precision medicine.

## Data Availability

The data supporting this study is available from the corresponding author upon request.

## Supplementary material

Supplementary Table 1 Populations included in the final dataset.

Supplementary Table 2 Genotypes included in the final dataset.

Supplementary Table 3 Genotype frequencies by region.

Supplementary Table 4 Exact p-values from pairwise testing.

Supplementary Figure 1 Global map of genotype frequencies in individual populations. Frequencies of *AA* (red), *CxTx* (dark green), *CxT4* (light green), *C4Tx* (dark blue), and *C4T4* (light blue) are shown as pie charts at their approximate geographic locations. Data points were slightly shifted to allow clear visualization of all populations.

Supplementary Figure 2 Results of the principal component analysis (PCA). Results for the analysis of AA, CxTx, CxT4, C4Tx and C4T4 are in panels A-F and results for Cen and Tel genotypes are in panels G-L. A,G) Relationships between principal components. B,H) Scree plot showing the contribution of each component. C,I) Table showing the importance of each component. D,J) Table showing the composition of the first three components. E,K) PCA plot with Cos² values of individual variables indicated by color. F,L) Cos² values of variables showing their contribution to the principal components.
