# Supplementary material for: Global patterns of KIR genotype diversity reflect human migration and regional differences
Source: Front Immunol. 2026 Aug 12;17:1902165. doi: 10.3389/fimmu.2026.1902165 (PMC13506890; doi:10.3389/fimmu.2026.1902165)

Supplement Figure 1

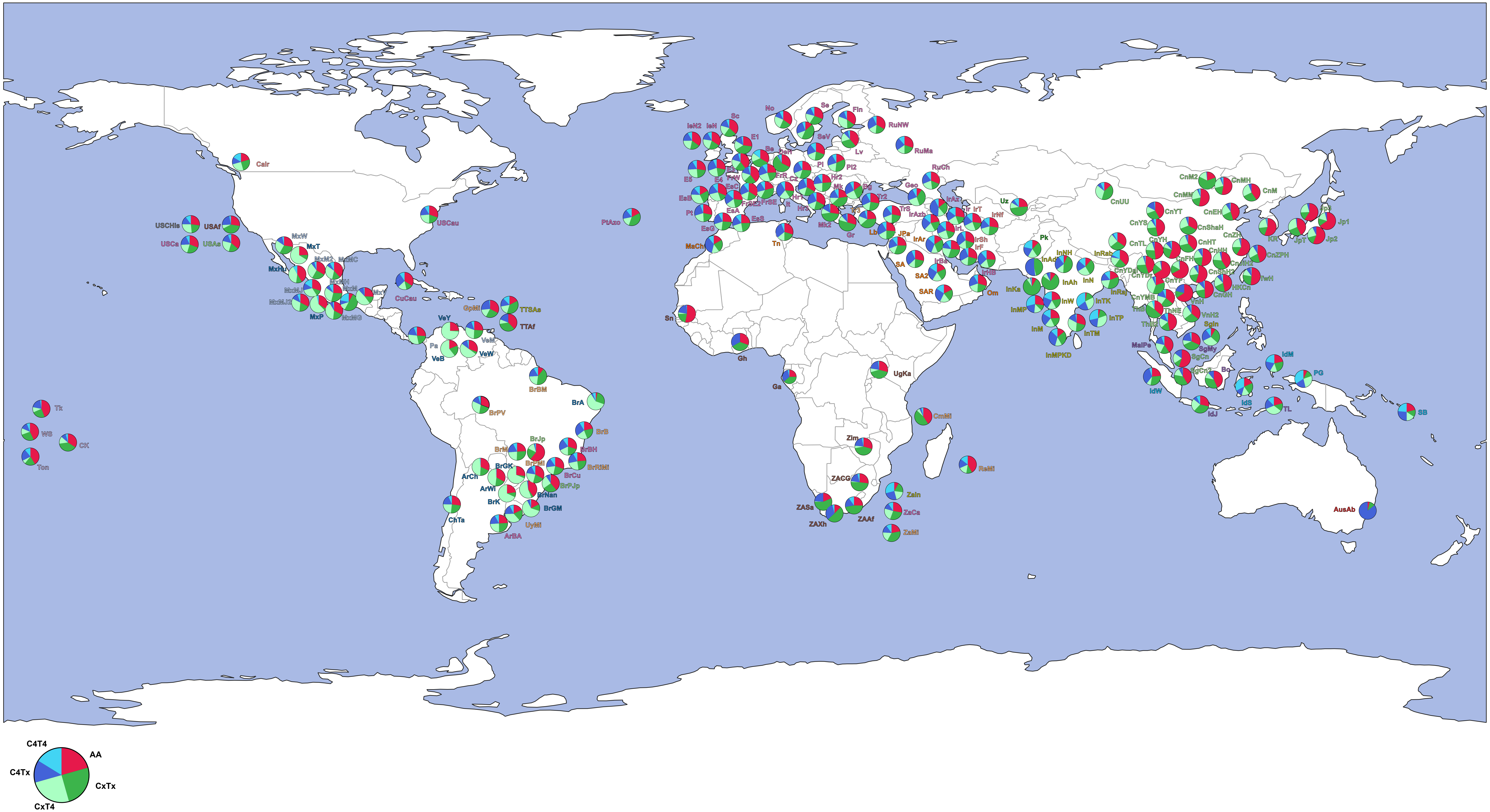

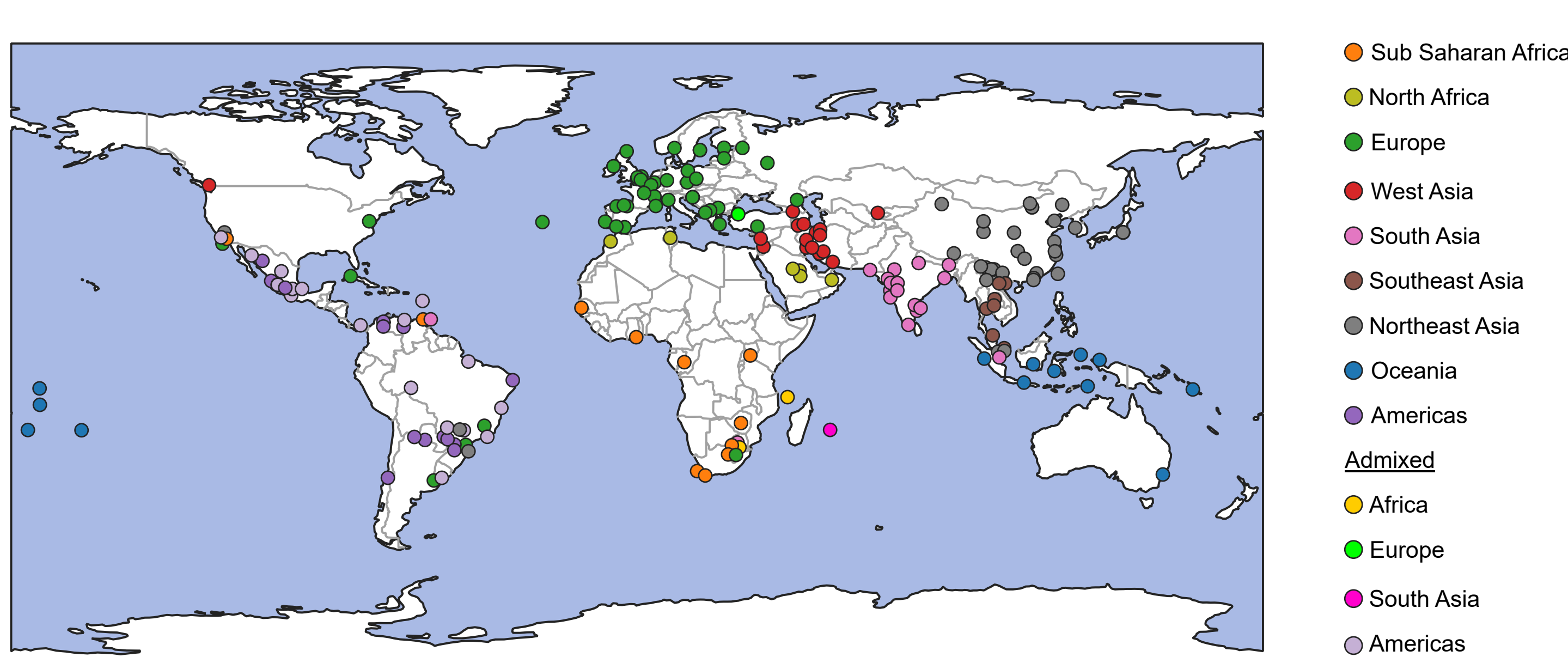

Supplement: Supplementary file 1 [file DataSheet1.pdf]
