# Supplementary material for: Global patterns of KIR genotype diversity reflect human migration and regional differences
Source: Front Immunol. 2026 Aug 12;17:1902165. doi: 10.3389/fimmu.2026.1902165 (PMC13506890; doi:10.3389/fimmu.2026.1902165)

Supplement Figure 2

A

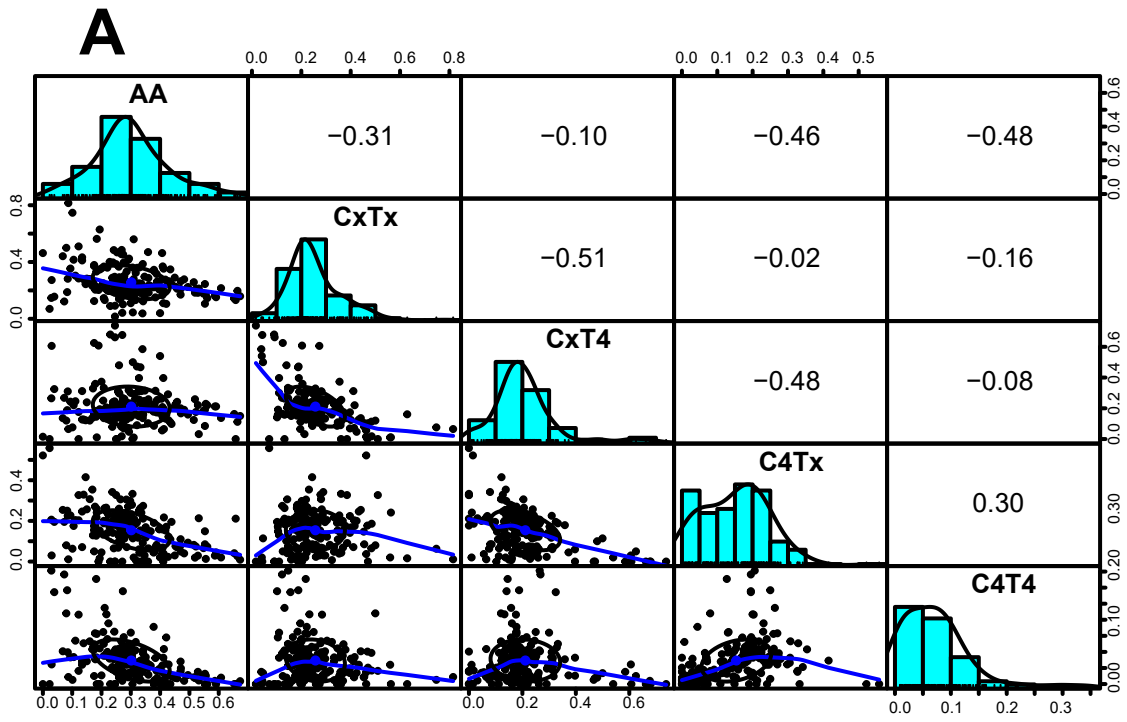

B

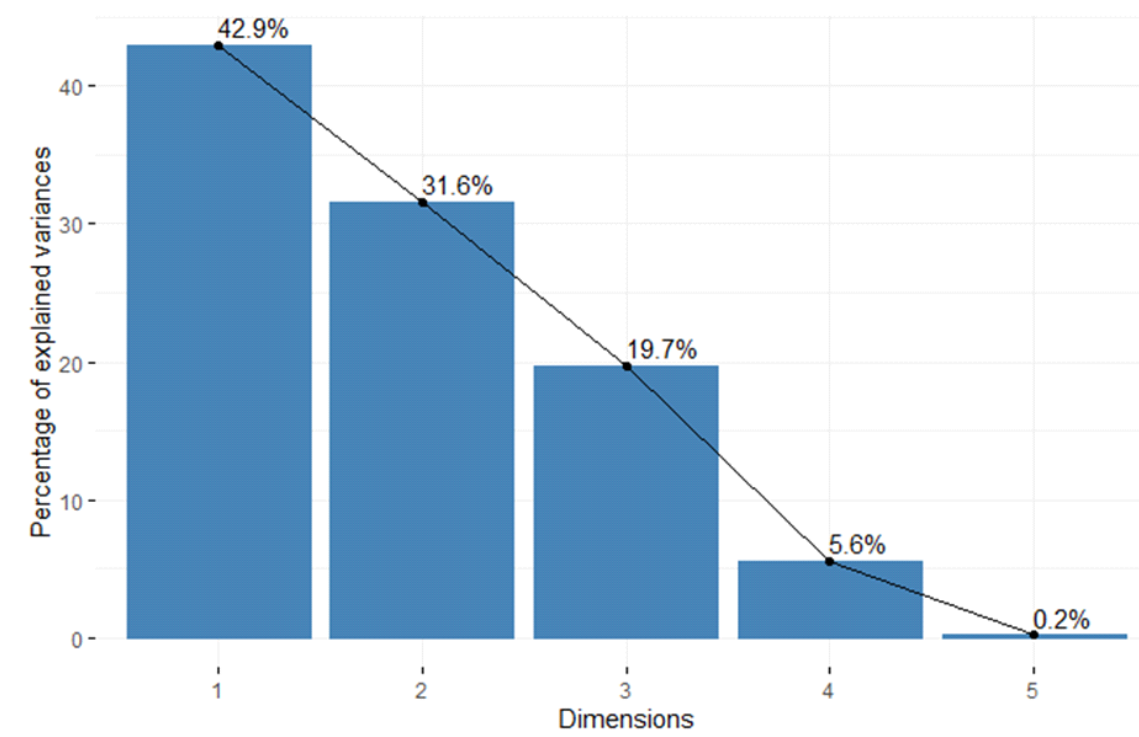

C

Importance of components:

	Comp.1	Comp.2	Comp.3	Comp.4	Comp.5
Standard deviation	0.1634743	0.1402893	0.1107236	0.05883630	0.01136880
Proportion of Variance	0.4292600	0.3161337	0.1969255	0.05560478	0.00207611
Cumulative Proportion	0.4292600	0.7453936	0.9423191	0.99792389	1.00000000

D

	Comp.1	Comp.2	Comp.3
AA	0.4490887	0.7650492	0.1346552
CxTx	-0.5376686	0.1813026	-0.6906915
CxT4	0.5972076	-0.5546493	-0.3038554
C4Tx	-0.3760402	-0.1656648	0.6061301
C4T4	-0.1056820	-0.2162137	0.2123474

E

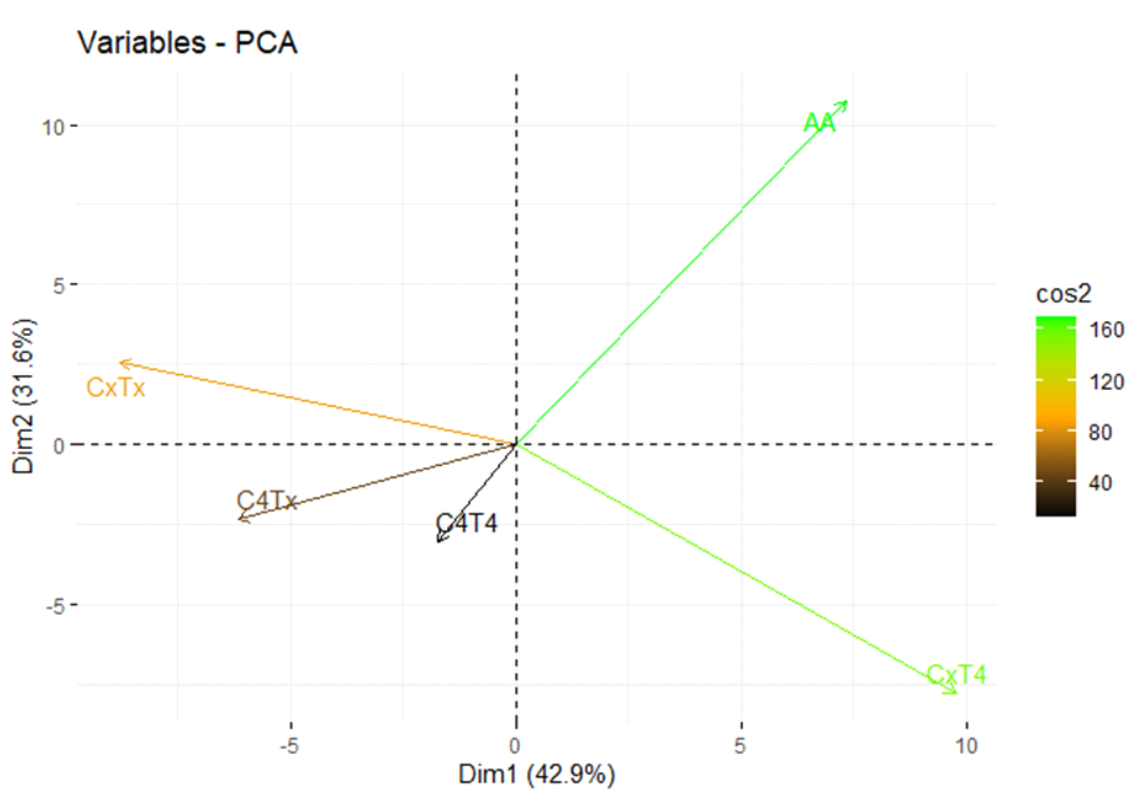

F

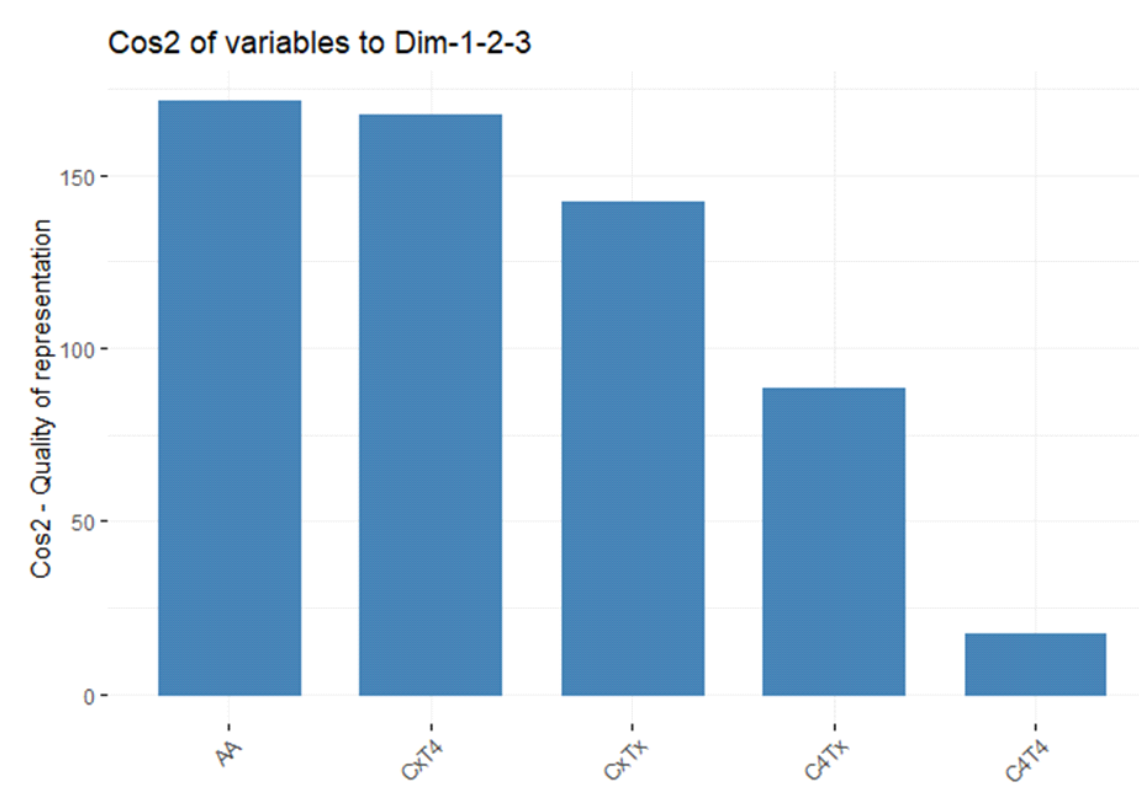

G

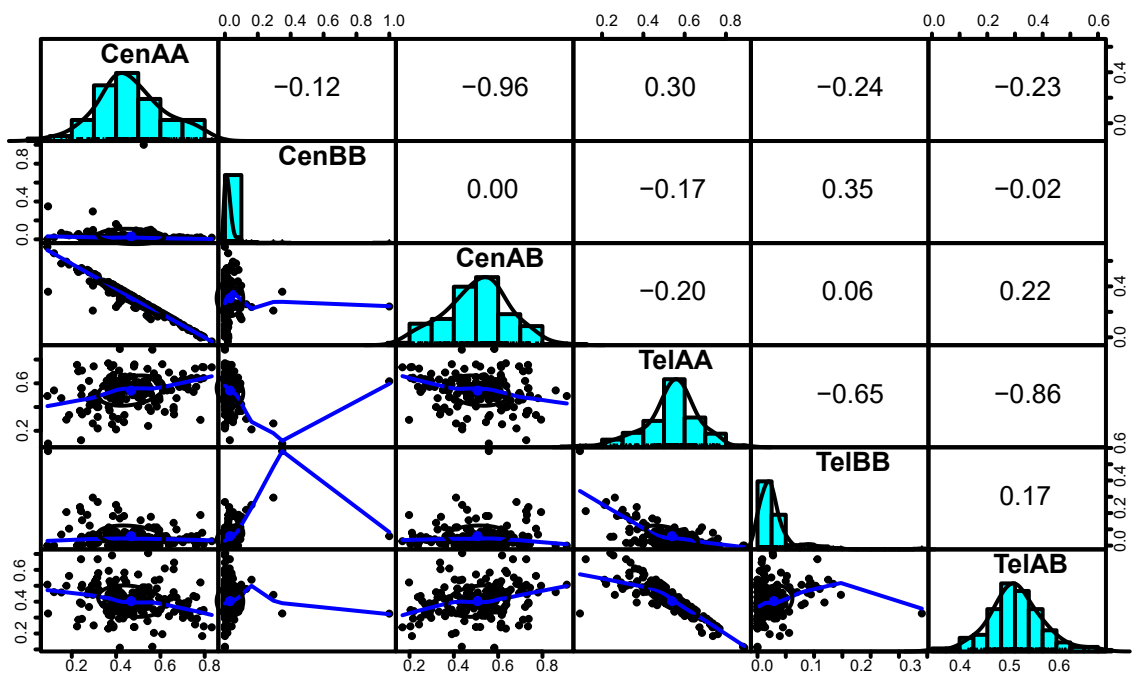

H

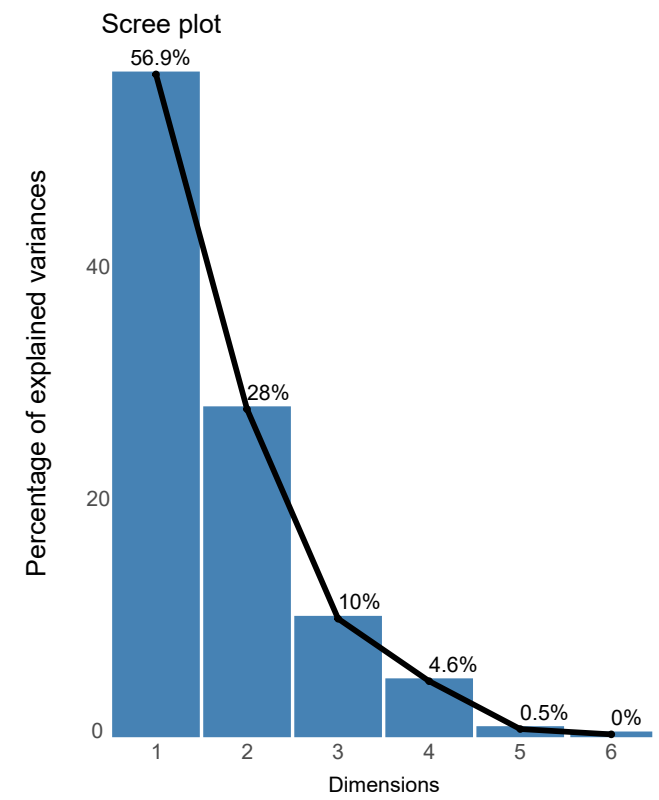

I

```
IImportance of components:
                          Comp.1    Comp.2     Comp.3     Comp.4      Comp.5
Standard deviation     0.2117016 0.1485990 0.08861708 0.06008213 0.018998604
Proportion of Variance 0.5693065 0.2804978 0.09975452 0.04585518 0.004585018
Cumulative Proportion  0.5693065 0.8498043 0.94955877 0.99541395 0.999998972
                             Comp.6
Standard deviation     2.844622e-04
Proportion of Variance 1.027891e-06
Cumulative Proportion  1.000000e+00
```

J

```
           Comp.1      Comp.2     Comp.3
CenAA  0.67632918  0.24347164  0.1257883
CenBB -0.04285538  0.07813065 -0.8052756
CenAB -0.62261560 -0.31521540  0.1421495
TelAA  0.31183399 -0.72698374  0.0351746
TelBB -0.09590670  0.21762979 -0.4136609
```

K

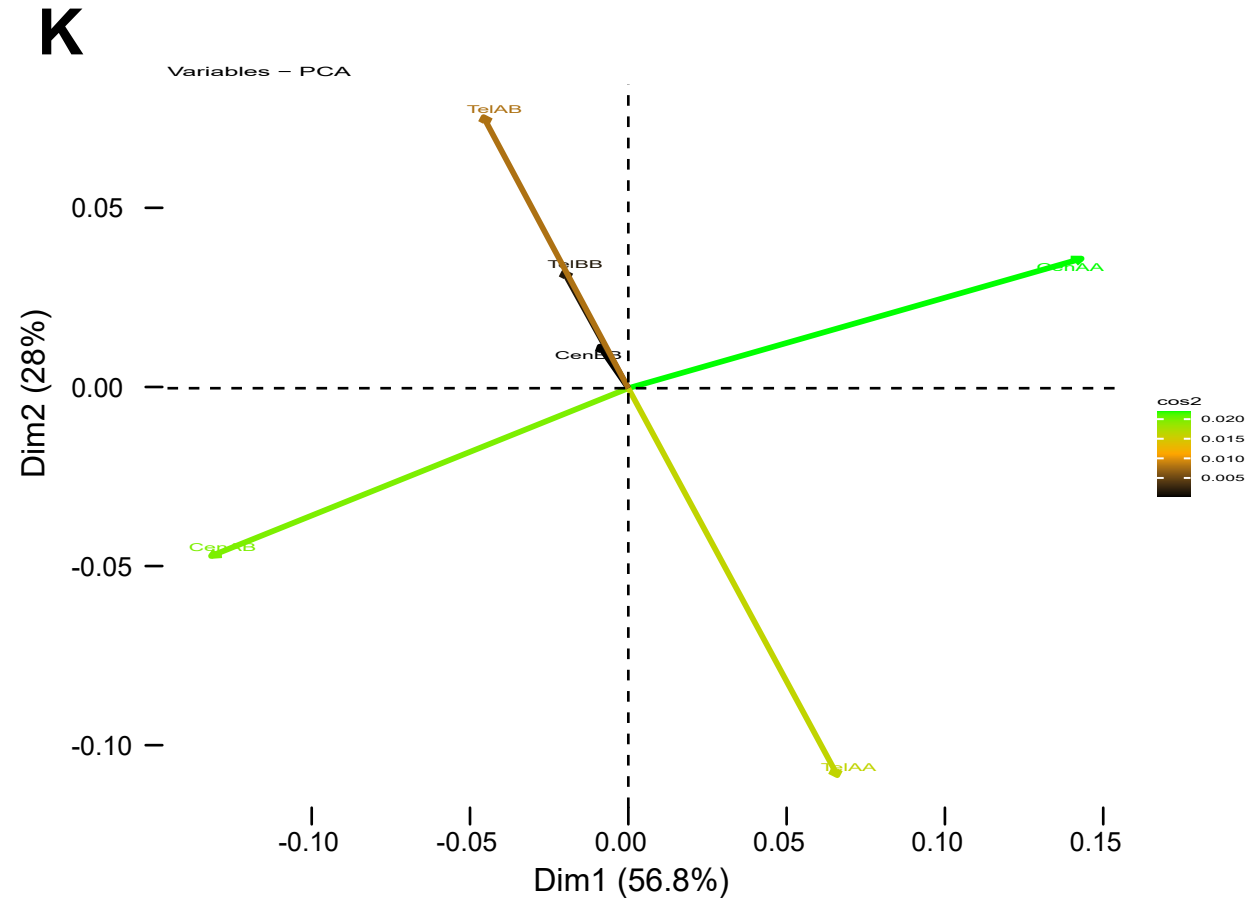

L

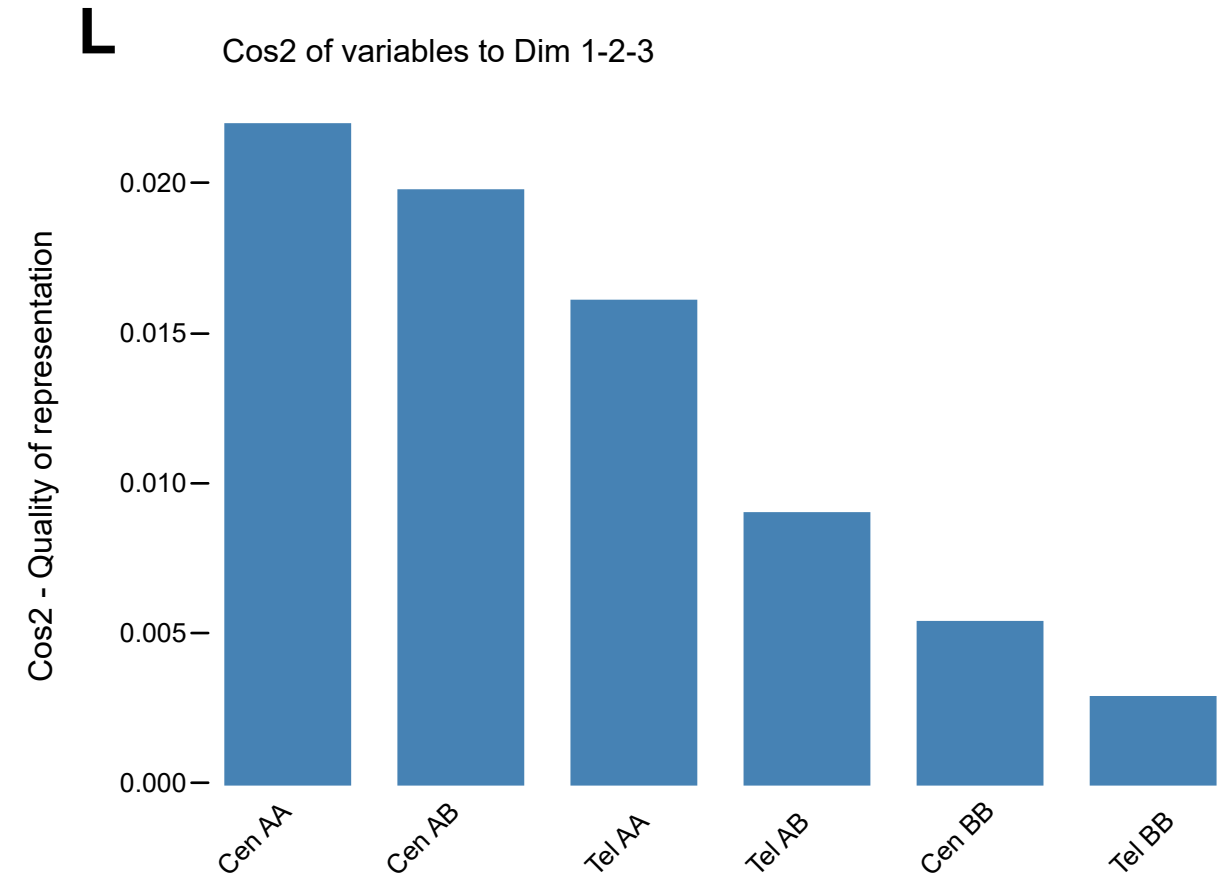

Supplement: Supplementary file 2 [file DataSheet2.pdf]
